# Supplementary material for: “I Solemnly Swear”: A Comparative Study of Codes of Professional Ethics amongst Pharmacists from Culturally Diverse European Countries
Source: Pharmacy (Basel). 2024 Sep 24;12(5):143. doi: 10.3390/pharmacy12050143 (PMC11510965; doi:10.3390/pharmacy12050143)
Supplement: Supplementary file 1 [file pharmacy-12-00143-s001.zip › pharmacy-3125036-supplementary.pdf]

Supplementary Material

Table S1. Listing of all the codes

A. Economic matters

**A.1. Market competition and advertisement**	**A.1.1. Allowed contents**	Determines what can be categorised as legal advertising and what constitutes prohibited marketing material.
	**A.1.2. Disloyal competition**	Prohibits possible actions that constitute a lie concerning the professional practice of other professionals or that advertise with false claims.
	**A.1.3. Prohibition**	A complete ban on advertising.
	**A.1.4. Truthfulness**	The materials disseminated must be based only on truthful content.
**A.2. Relationship with the pharmaceutical industry**	**A.2.1. Accepted material offerings**	Possible exceptions to the bribery definition; what can be classified as a non-bribery situation.
	**A.2.2. Refusal of bribes**	Pharmacists must not accept payments or incentives from the pharmaceutical industry that could mislead them and unethically alter the health services they provide.

B. Genetic ethics

**B.1. Genetic modification**	**B.1.1. When allowed**	In which cases can genetic modification be authorised.
**B.2. Pharmacogenetics**	**B.2.1. Subsistence**	Mention the existence of pharmacogenetics and the reality of its application in the current era of pharmacological development.
	**B.2.2. Justified aims**	When the use of pharmacogenetics in favour of better public health is justified and permitted.

C. Pharmacists-Patient relationship

**C.1. Patient data**	**C.1.1. Confidentiality**	**C.1.1.1. Applications**	What constitutes confidential information.
		**C.1.1.2. Respect and protection (FIP)**	Respect and protection of the confidentiality of patient information.
		**C.1.1.3. Compliance with the law (FIP)**	All patient data collected must comply with the applicable laws on the protection and retention of patient data.
		**C.1.1.4. Informed consent (FIP)**	The patient's informed consent is required for the processing, publication or disclosure of information covered by the confidentiality of the patient-pharmacist relationship.
	**C.1.2. Patient registry**	**C.1.2.1. Definition**	What do patient registers consist of, and what is their purpose.
		**C.1.2.2. Protection from hacking**	Obligation to take measures to prevent third parties from accessing patient data.
**C.2. Moral relationship**	**C.2.1. Patient dignity**		Respecting the patient as an autonomous person, protecting his privacy and offering emotional support and honesty
	**C.2.2. Patient autonomy**		Patients must be able to make choices and have the right to be informed so they can make informed choices.
**C.3. Spirit of Collaboration**	**C.3.1. With patients and carers (FIP)**		The pharmacist should cooperate with the patient or, in cases where the patient needs support, with the carer.
**C.4. Cultural differences and conflict**	**C.4.1. Respecting divergence**	**C.4.1.1. Patient (FIP)**	The pharmacist must not discriminate against the patient based on personal or lifestyle characteristics or allow personal views to influence care.

		**C.4.1.2. Careguivers (FIP)**	The pharmacist must not discriminate against the patient's carer on the basis of personal or lifestyle characteristics or allow personal views to influence care.
		**C.4.1.3. Continuation of treatment (FIP)**	In the event of a conflict, the pharmacist should ensure continuity of care for the patient, whether the patient is directly or indirectly involved. In the case of conflict with caregivers or other health professionals, the patient can be assumed to be indirectly involved because the outcomes of the conflict affect him or her, even if the patient is not directly involved.
**C.5. Healthcare for specific groups**	**C.5.1. Healthcare services to detainees**		Pharmacists must guarantee appropriate healthcare to detainees.

D. Relationship with the National Pharmaceutical Society

**D.1. Rights**	**D.1.1. Delegation of conflict**	In case of conflicts between pharmacists, the pharmaceutical society can act as a moderator and help to settle disputes.
	**D.1.2. Exclusivity**	Only pharmacists (who are registered in the pharmaceutical society) can open pharmacies.
	**D.1.3. Legal representation**	Pharmacists have the right to legal representation supported by their national pharmaceutical society in cases concerning their practice.
	**D.1.4. Professional identity**	Members have the right to call themselves pharmacists because they have a university education in pharmaceutical sciences and are members of the pharmaceutical society of their country.

	**D.1.5. Other**	Other possible rights guaranteed by the respective national pharmaceutical society that are not already included in the above points.
**D.2. Duties**	**D.2.1. Confidentiality**	Professional regulation may classify some information as confidential; pharmacists must respect this confidentiality and not disclose the information to the public.
	**D.2.2. Reporting**	The reporting of violations of the law or other acts affecting the ethical and deontological aspects of the pharmaceutical profession.

E. Professional positioning

**E.1. Professional attitude**	**E.1.1. Values**	**E.1.1.1. Honesty (FIP)**	Honesty constitutes fair and straightforward behaviour that comes from a refusal to lie.
		**E.1.1.2. Integrity (FIP)**	Character trait of remaining true to moral values by applying them in daily life and standing up for them when necessary.
		**E.1.1.3. Professionalism (FIP)**	Combination of attributes and competencies associated with a particular professional practice. These include knowledge, skills, behavioural qualities and moral values.
		**E.1.1.4. Trustworthiness (FIP)**	Trust in another person's moral character and competence. It entails confidence that the other will act with the right motives and according to appropriate moral standards.

	**E.1.2. Legal correctness**	**E.1.2.1. Legislation and recognised codes**	**E.1.2.1.1. Need for compliance (FIP)**	Pharmacists must comply with the national and international regulations of the country in which they operate. This may include documents adopted by the pharmaceutical society of the respective country, provided that these bodies have legal powers.
**E.2. Professional priorities**	**E.2.1. Safety and welfare (FIP)**			Pharmacists must guarantee that the safety and well-being of their patients is a priority and a mandatory requirement.
	**E.2.2. Healthcare quality (FIP)**			Ensure that the best possible quality of healthcare is provided
	**E.2.3. Equity (FIP)**			The principle whereby everyone is treated fairly according to their needs.
	**E.2.4. Justice (FIP)**			Meeting the population's needs, in their plenitude of right, while balancing economic and social factors such as increases in the cost of treatment and ageing.
	**E.2.5. Resource managing (FIP)**			Recognizing the limited nature of resources and optimally managing these resources.
	**E.2.6. Sanitation and sustainability**	**E.2.6.1. Hygiene and the importance of clean spaces**		The importance of complying with the required cleaning and safety requirements to ensure the safety of colleagues and patients.

		**E.2.6.2. Sustainability and ecological matters**	The need for ecological awareness and the minimisation of disposable waste.
	**E.2.7. Veterinary matters**	**E.2.7.1. Veterinary health and medicine**	The collaboration of pharmacists with veterinarians and the importance of maintaining and protecting animal health and life.
	**E.2.8. Pharmaceutical intervention**	**E.2.8.1. Health promotion**	Pharmacists should support health promotion ideas and promote patient health literacy.
		**E.2.8.2. Rational use of medicines**	Ensuring that the various therapeutics available are only used when they are needed and are given to the right person.
**E.3. Competencies**	**E.3.1. Managing of competencies and leadership**		Pharmacists need to be aware of their skills and limitations in their work and, simultaneously, recognize the skills and limitations of other pharmacists and health professionals to lead and work together.
	**E.3.2. Limitations**	**E.3.2.1. Dependency on doctors**	Pharmacists cannot change doctors' prescriptions or administer medicines to patients.
		**E.3.2.2. Refrain from diagnosing**	Pharmacists are not allowed to diagnose or advise patients on the nature of the disease or whether or not they agree with the physician

**E.4. Professional role identity**	**E.4.1. Professional development**	**E.4.1.1. Continous formation (FIP)**	Pharmacists should constantly improve their scientific knowledge and keep up with the latest innovations in health and science.
		**E.4.1.2. Mentoring**	Pharmacists should take an active role in the formation of future professionals.
	**E.4.2. Laboral rights**	**E.4.2.1 Fair wage**	Pharmacists should strive and ensure they receive a fair wage for their services and apply the same standards when employing other pharmacists.
		**E.4.2.2. Non-discrimination**	The pharmacist shall not be discriminated against based on a belief or personal practice.
		**E.4.2.3. Professional dignity**	Based on self-respect and self-confidence. Resilience to abuse, pride in daily work, a sense of purpose, and camaraderie and solidarity with colleagues.
		**E.4.2.4. Strikes**	Pharmacists have the right to organise and participate in strikes.
	**E.4.3. Professional autonomy**	**E.4.3.1. Recognition**	Reflection on the privilege of self-regulation with subsequent concretisation of this right in a code of ethics
		**E.4.3.2. Possible existence of divided loyalties**	Possible conflict between the pharmacist's moral obligation to patients and the need to

				maintain the pharmacy as a business.
	**E.4.4. Moral positioning**	**E.4.4.1. Moral objection**	**E.4.4.1.1. Clinical objection**	Pharmacists may refuse to perform a certain action because they believe that this intervention may harm or worsen the patient's health.
			**E.4.4.1.2. As a pharmacist right**	The pharmacist has the right to refuse to perform a specific act required by law based on their moral convictions
		**E.4.4.2. War and conflict**	**E.4.4.2.1. Refusal to participate in torture or war crimes**	In the event of war or conflict, pharmacists must refuse to participate in acts that diminish human life, such as torture or war crimes.
			**E.4.4.2.2. Role in case of an event**	Pharmacists must co-operate and work together with the various healthcare professionals. They must not allow personal views and conflicts to influence patient care

F. Interprofessional cooperation

**F.1. Spirit of collaboration**	**F.1.1. With healthcare industries (FIP)**		Pharmacists need to cooperate and collaborate with the different healthcare industries
	**F.1.2. With other healthcare professionals (FIP)**		Pharmacists need to cooperate and collaborate with other healthcare professionals
	**F.1.3. With other pharmacists (FIP)**		Pharmacists need to cooperate and collaborate with other pharmacists.
**F.2. Cultural differences and conflict**	**F.2.1. Respecting divergence**	**F.2.1.1. Healthcare professionals (FIP)**	The pharmacist mustn't discriminate against other healthcare professionals based on any personal or lifestyle traits or allow personal views and conflicts to influence the care provided to the patient.

Figure S1. Mind map of total coding used in each code

A. Portugal

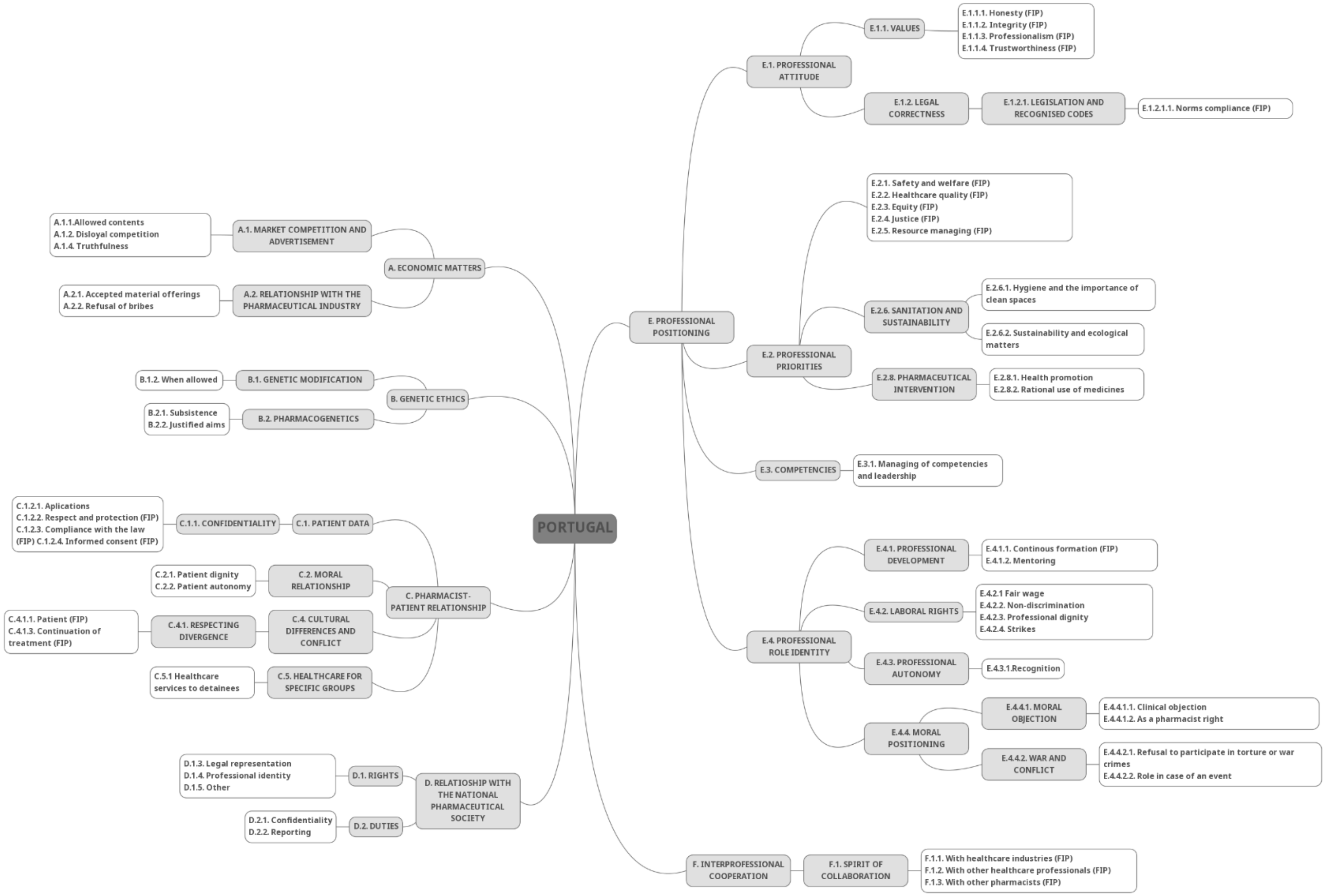

B. Lithuania

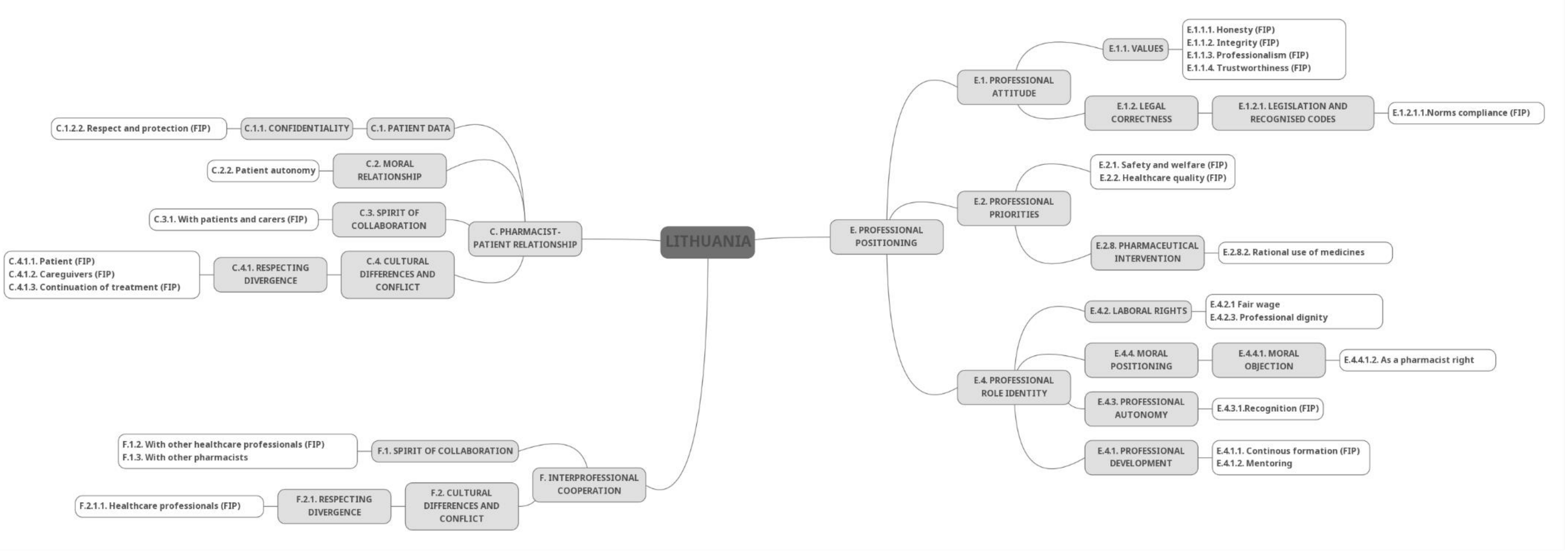
LITHUANIA
C. PHARMACIST-PATIENT RELATIONSHIP
C.1. PATIENT DATA
C.1.1. CONFIDENTIALITY
C.1.2.2. Respect and protection (FIP)
C.2. MORAL RELATIONSHIP
C.2.2. Patient autonomy
C.3. SPIRIT OF COLLABORATION
C.3.1. With patients and carers (FIP)
C.4. CULTURAL DIFFERENCES AND CONFLICT
C.4.1. RESPECTING DIVERGENCE
C.4.1.1. Patient (FIP)
C.4.1.2. Careguivers (FIP)
C.4.1.3. Continuation of treatment (FIP)
F. INTERPROFESSIONAL COOPERATION
F.1. SPIRIT OF COLLABORATION
F.1.2. With other healthcare professionals (FIP)
F.1.3. With other pharmacists
F.2. CULTURAL DIFFERENCES AND CONFLICT
F.2.1. RESPECTING DIVERGENCE
F.2.1.1. Healthcare professionals (FIP)
E. PROFESSIONAL POSITIONING
E.1. PROFESSIONAL ATTITUDE
E.1.1. VALUES
E.1.1.1. Honesty (FIP)
E.1.1.2. Integrity (FIP)
E.1.1.3. Professionalism (FIP)
E.1.1.4. Trustworthiness (FIP)
E.1.2. LEGAL CORRECTNESS
E.1.2.1. LEGISLATION AND RECOGNISED CODES
E.1.2.1.1.Norms compliance (FIP)
E.2. PROFESSIONAL PRIORITIES
E.2.1. Safety and welfare (FIP)
E.2.2. Healthcare quality (FIP)
E.2.8. PHARMACEUTICAL INTERVENTION
E.2.8.2. Rational use of medicines
E.4. PROFESSIONAL ROLE IDENTITY
E.4.2. LABORAL RIGHTS
E.4.2.1 Fair wage
E.4.2.3. Professional dignity
E.4.4. MORAL POSITIONING
E.4.4.1. MORAL OBJECTION
E.4.4.1.2. As a pharmacist right
E.4.3. PROFESSIONAL AUTONOMY
E.4.3.1.Recognition (FIP)
E.4.1. PROFESSIONAL DEVELOPMENT
E.4.1.1. Continous formation (FIP)
E.4.1.2. Mentoring

C. Turkey

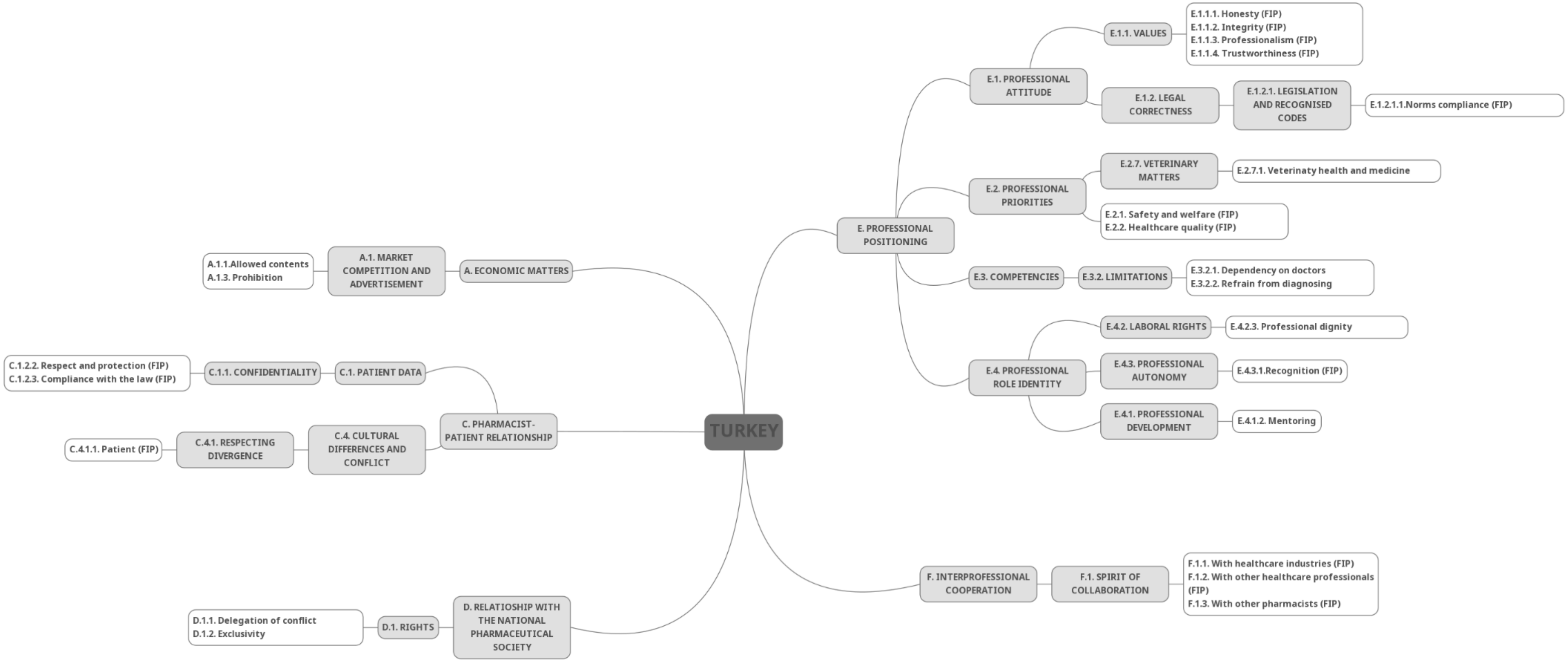

TURKEY
A. ECONOMIC MATTERS
A.1. MARKET COMPETITION AND ADVERTISEMENT
A.1.1.Allowed contents
A.1.3. Prohibition
C. PHARMACIST-PATIENT RELATIONSHIP
C.1. PATIENT DATA
C.1.1. CONFIDENTIALITY
C.1.2.2. Respect and protection (FIP)
C.1.2.3. Compliance with the law (FIP)
C.4. CULTURAL DIFFERENCES AND CONFLICT
C.4.1. RESPECTING DIVERGENCE
C.4.1.1. Patient (FIP)
D. RELATIOSHIP WITH THE NATIONAL PHARMACEUTICAL SOCIETY
D.1. RIGHTS
D.1.1. Delegation of conflict
D.1.2. Exclusivity
E. PROFESSIONAL POSITIONING
E.1. PROFESSIONAL ATTITUDE
E.1.1. VALUES
E.1.1.1. Honesty (FIP)
E.1.1.2. Integrity (FIP)
E.1.1.3. Professionalism (FIP)
E.1.1.4. Trustworthiness (FIP)
E.1.2. LEGAL CORRECTNESS
E.1.2.1. LEGISLATION AND RECOGNISED CODES
E.1.2.1.1.Norms compliance (FIP)
E.2. PROFESSIONAL PRIORITIES
E.2.7. VETERINARY MATTERS
E.2.7.1. Veterinaty health and medicine
E.2.1. Safety and welfare (FIP)
E.2.2. Healthcare quality (FIP)
E.3. COMPETENCIES
E.3.2. LIMITATIONS
E.3.2.1. Dependency on doctors
E.3.2.2. Refrain from diagnosing
E.4. PROFESSIONAL ROLE IDENTITY
E.4.2. LABORAL RIGHTS
E.4.2.3. Professional dignity
E.4.3. PROFESSIONAL AUTONOMY
E.4.3.1.Recognition (FIP)
E.4.1. PROFESSIONAL DEVELOPMENT
E.4.1.2. Mentoring
F. INTERPROFESSIONAL COOPERATION
F.1. SPIRIT OF COLLABORATION
F.1.1. With healthcare industries (FIP)
F.1.2. With other healthcare professionals (FIP)
F.1.3. With other pharmacists (FIP)

Table S2. Examples of original sentences and their translation in the Turkish and Lithuanian codes

Original text (Lithuanian)	Translated version (English)
Sąžiningai ir etiškai elgtis su savo kolegomis, kitais sveikatos priežiūros specialistais bei pacientais ar jų globėjais, puoselėjant visuomenės pasitikėjimą vaistininko profesija.	To act honestly and ethically towards colleagues, other healthcare professionals and patients or their carers, promoting public confidence in the profession of pharmacy.
Vaistininkas gerbia ir saugo farmacinės paslaugos metu gautos ir pateiktos informacijos konfidencialumą ir užtikrina jos panaudojimą teisės aktų nustatyta tvarka.	The pharmacist shall respect and protect the confidentiality of the information obtained and provided in the course of the pharmaceutical service and shall ensure that it is used following the legal provisions.
Vaistininkas dirba bendradarbiaudamas su visa vaistinės komanda ir deda visas pastangas, siekdamas išvengti bet kokių klaidų vaistinės darbe.	The pharmacist works in cooperation with the whole pharmacy team and makes every effort to avoid any mistakes in pharmacy work.
Atsižvelgiant į šiai profesijai tenkančią atsakomybę ir kvalifikacinius reikalavimus, vaistininkas turi teisę į saugias darbo sąlygas, orų atlyginimą bei sudarytas palankias galimybes savo kvalifikacijai kelti.	Pharmacists shall have the right to safe working conditions, decent remuneration and favourable opportunities for the development of their skills, taking into account the responsibilities and qualifications of the profession.

Original text (Turkish)	Translated version (English)
Eczacının başta gelen görevi, birbirleriyle hekim ve veteriner hekimlerle tam bir anlayış ve işbirliği içinde çalışarak insan ve hayvan sağlığına, hayatına ihtimam göstermektir.	The primary task of the pharmacist is to ensure full understanding and cooperation with physicians and veterinary to take care of human and animal health.
Eczacı, reçeteyi yazan hekim, reçetede adı yazılı hasta veya reçeteyi getiren kişi kim olursa olsun; cinsiyet, dil ırk, milliyet, felsefi inanç, din ve mezhep, ahlaki düşünce, karakter ve kişilik, toplumsal seviye, mevki ve siyasi düşünce ayırımı yapmaksızın ilacını hazırlama ve reçete sahibine verme hususunda azami dikkat ve ihtimamı göstermekle yükümlüdür.	Regardless of the prescribing physician, the patient whose name is written on the prescription or the person who brings the prescription, the pharmacists must show the utmost care and care in preparing the medicine and giving it to the prescriber, without discrimination, regardless of gender, language, race, nationality, philosophical belief, religion and sect, moral opinion, character and personality, social level, position and political beliefs.
Eczacı, staj yapan öğrencilerin, eczanenin faaliyetine, özellikle laboratuvar çalışmalarına katılarak iyi yetişmelerini, meslek sevgi, saygı ve ruhunun yerleşmesini sağlamaya çalışır.	The pharmacist shall ensure that the internship students participate in the activities of the pharmacy, especially in laboratory work, to ensure their upbringing and the establishment of professional integrity, respect and spirit.

Eczacı, Türk Eczacıları Birliğinin bu Tüzük hükümleri dahilinde aldığı her türlü deontoloji kararları ile eczanelerin açılma ve kapanma saatleri, tatil günleri ve eczane nöbetleri hakkında yetkili makamlarca verilen kararlara uymak zorundadır.	The pharmacist shall comply with all kinds of deontological decisions of the Turkish Pharmacists Association within the provisions of this regulation and the decisions made by the competent authorities regarding the opening and closing hours of pharmacies, holidays and pharmacy shifts.
